# Measuring Linewidth Enhancement Factor by Relaxation Oscillation Frequency in a Laser with Optical Feedback

**DOI:** 10.3390/s18114004

**Published:** 2018-11-16

**Authors:** Yuxi Ruan, Bin Liu, Yanguang Yu, Jiangtao Xi, Qinghua Guo, Jun Tong

**Affiliations:** School of Electrical, Computer and Telecommunications Engineering, University of Wollongong, Northfields Avenue, Wollongong, NSW 2522, Australia; yr776@uowmail.edu.au (Y.R.); bl987@uowmail.edu.au (B.L.); jiangtao@uow.edu.au (J.X.); qguo@uow.edu.au (Q.G.); jtong@uow.edu.au (J.T.)

**Keywords:** linewidth enhancement factor, relaxation oscillation, external optical feedback, self-mixing interferometry, laser sensors

## Abstract

This paper presents a new method for measuring the linewidth enhancement factor (alpha factor) by the relaxation oscillation (RO) frequency of a laser with external optical feedback (EOF). A measurement formula for alpha is derived which shows the alpha can be determined by only using the RO frequencies and no need to know any other parameters related to the internal or external parameters associated to the laser. Unlike the existing EOF based alpha measurement methods which require an external target has a symmetric reciprocate movement. The proposed method only needs to move the target to be in a few different positions along the light beam. Furthermore, this method also suits for the case with alpha less than 1. Both simulation and experiment are performed to verify the proposed method.

## 1. Introduction

Semiconductor lasers (SL) due to their small size, large gain per unit length and wide gain spectrum play a key role in the emerging field of optoelectronics, such as optical sensor, optical communication and optical disc system, etc. Henry in 1982 found SLs exhibit a strong variation of refractive index and gain when the injected carrier density is changed, he introduced the linewidth enhancement factor (also called α factor) to describe this dependence and it is defined as $\alpha =(\partial n_{R}/\partial N)/(\partial n_{I}/\partial N)$, where $N,\;n_{R}$ and $n_{I}$ are, respectively, the carrier density, the real and imagery part of the refractive index [1]. The α factor is regarded as a fundamental descriptive parameter of the SL. It characterizes the characteristics of SLs such as the spectral effects, the modulation response, the injection locking and the response to the external optical feedback [2]. Therefore, an accurate value of the α factor is vital for behaviour analysis of an SL and designing its application systems. Various techniques have been explored for measuring the α factor. These techniques can be mainly classified [3] as: (1) The direct linewidth measurement [4]; (2) The current modulation [5]; (3) The optical injection [6] and (4) The optical feedback technique [7,8,9,10]. Among all these techniques, optical feedback technique is an emerging and promising method reflecting a minimum part-count scheme, which provides an ease of implementation and simplicity in system configuration [11,12,13,14].

**Figure 1:**
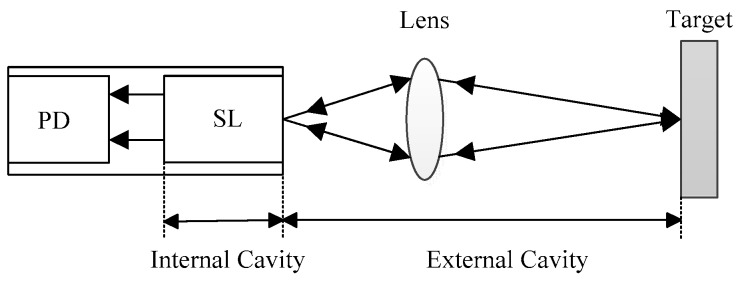
Schematic configuration of an SMI.

The optical feedback technique is based on the self-mixing effect. It occurs when a small fraction of the light emitted by an SL is back reflected or back scattered by a remote target and re-enters the laser cavity [15,16]. In this case, the steady-state intensity of the lasing light is modulated due to a varying external optical feedback phase. The modulated SL steady-state intensity is considered as a self-mixing signal which carries the information of the parameters related to the SL and its external cavity [7,17]. A basic configuration of a self-mixing interferometry (SMI) is shown in Figure 1, which consists of an SL, a photodiode (PD) packaged in the rear of the SL, a lens and external target. The front facet of the SL and the target form the external cavity.

Based on the SMI configuration, various methods have been presented for the measurement of α factor. In 2004, Yu et al. [7] proposed a method to measure α factor by using the hysteresis of SMI signals when the external target has a reciprocating movement. However, this method needs the SMI system being in moderate feedback regime and SMI signals have zero-crossing points, i.e., the optical feedback level (denoted by C) in the range of 1 < C < 3. The required condition on the feedback level may not be met in some practical applications. Although an attenuator can be used to adjust the optical feedback to the measurement range with 1 < C < 3, however this may lead to a larger measurement error as α factor is influenced by optical feedback level [10]. Then several other SMI-based methods were developed [8,9,10,18,19] for covering different C level, e.g., the methods in [8,9] are for 0 < C < 1 and methods in [10,18] for 1 < C < 4.6. These methods for retrieving α are based on commonly accepted SMI waveform model by further data processing applied on the waveform of a self-mixing signal, e.g., phase unwrapping in [10,19], and data-to-model fitting algorithms in [8,9,18]. However, the reported hysteresis and it resulted sawtooth-like SMI waveform is on the case with α>1 [7,10,20]. For α<1, the hysteresis in an SMI waveform does not follow the switching law reported in [7,20]. Thus, the existing algorithms developed for α cannot work. To fill the gap, it requires to develop a method which does not rely on the waveform of an SMI signal.

Recently, sensing and measurement using dynamics of the laser with optical feedback have been reported [21,22]. High sensing sensitivity by using the relaxation oscillation (RO) frequency has been demonstrated [21]. This gives us an inspiration to measure α factor by laser dynamics.

In this work, a method based on the laser dynamics for measuring α factor is presented. Staring from the well-known Lang–Kobayashi (L–K) equations, we propose to measure α factor using the RO frequency of an SL with optical feedback. Then both simulations and experiments are conducted to verify the feasibility of the proposed method. Additionally, the proposed method also provides a way to measure small values of α.

## 2. Measurement Theory

The dynamics of an SL with optical feedback can be described by Lang and Kobayashi (L–K) equations [23]. Three variables, electric field amplitude E(t), electric field phase ϕ(t), carrier density N(t) and other parameters are associated with SL and its external cavity are shown in Equations (1)–(3)

(1) $$\frac{dE(t)}{dt}=\frac{1}{2}\left\{G\left[N(t),E(t)\right]-\frac{1}{\tau_{p}}\right\}E(t)+\frac{\kappa }{\tau_{in}}\cdot E(t-\tau )\cdot \cos\left[\omega_{0}\tau +\phi (t)-\phi (t-\tau )\right]\;$$

(2) $$\frac{d\phi (t)}{dt}=\frac{1}{2}\alpha \left\{G\left[N(t),E(t)\right]-\frac{1}{\tau_{p}}\right\}-\frac{\kappa }{\tau_{in}}\cdot \frac{E(t-\tau )}{E(t)}\cdot \sin\left[\omega_{0}\tau +\phi (t)-\phi (t-\tau )\right]\;$$

(3) $$\frac{dN(t)}{dt}=J-\frac{N(t)}{\tau_{s}}-G\left[N(t),E(t)\right]E^{2}(t)\;$$

where $G\left[N(t),E(t)\right]=G_{N}\left[N(t)-N_{0}\right]\left[1-\varepsilon \Gamma E^{2}(t)\right]$ is the modal gain per unit time, please note the nonlinear gain term is ignored in this work. The physical meanings of the symbols appearing in Equations (1)–(3) and the values of the parameters used in the simulations of this paper are shown in Table 1 [20]. The laser intensity is calculated by $I\left(t\right)=E^{2}\left(t\right)$.

The widely accepted mathematical model for describing an SMI waveform is derived from the steady state solutions of the L-K equations by setting dE(t)/dt=0, $d\phi (t)/dt=\omega_{s}-\omega_{0}$ and dN(t)/dt=0. The model consists of the following [20]:

(4) $$\omega_{s}\tau =\omega_{0}\tau -C\sin(\omega_{s}\tau +\arctan\alpha )\;$$

(5) $$N_{s}=N_{0}+\frac{1}{G_{N}\tau_{p}}-\frac{2\kappa \cos(\omega_{s}\tau )}{\tau_{in}G_{N}}\;$$

(6) $$E_{s}^{2}=\frac{\left(J-N_{s}/\tau_{s}\right)}{G_{N}(N_{s}-N_{0})}\;$$

Equation (4) is called the phase equation, where $\omega_{s}\tau$ and $\omega_{0}\tau$ are the light phase with and without feedback respectively. The laser output power or intensity is $E_{s}^{2}$, denoted by $P=E_{s}^{2}$, which can be expressed as: $P\;=\;P_{0}+\Delta P$, where $P_{0}$ is the laser output power without feedback, ΔP is the variation part due to optical feedback. By substituting Equation (5) into Equation(6), the normalized variation of the SL output power (denoted by $\Delta P/\Delta P_{\max}$) also called the SMI signal can be described as

(7) $$\Delta P/\Delta P_{\max}=\cos(\omega_{s}\tau )\;$$

The relaxation oscillation frequency (denoted as $f_{RO}$) of the SL can be obtained by linear stability analysis for the system described by L–K equations. With the conditions of $\kappa \tau /\tau_{in}<<1$, an expression for $f_{RO}$ can be derived from L–K equations given as below [24]

(8) $$f_{RO}=\frac{1}{2\pi }\sqrt{\frac{J-1}{\tau_{s}\tau_{p}}\left(1+G_{N}N_{0}\tau_{p}\right)}\sqrt{\frac{\left[1-2\frac{\kappa }{\tau_{in}}\tau_{p}\cos\left(\omega_{s}\tau \right)\right]\left[1+\frac{\kappa }{\tau_{in}}\tau \cos\left(\omega_{s}\tau \right)-\alpha \frac{\kappa }{\tau_{in}}\tau \sin\left(\omega_{s}\tau \right)\right]}{1+{\left(\frac{\kappa }{\tau_{in}}\right)}^{2}\tau^{2}+2\frac{\kappa }{\tau_{in}}\tau \cos\left(\omega_{s}\tau \right)}}\;$$

where $\frac{1}{2\pi }\sqrt{\frac{J-1}{\tau_{s}\tau_{p}}\left(1+G_{N}N_{0}\tau_{p}\right)}$ is the RO frequency of a solitary SL (denoted by $f_{RO-zero}$). $\omega_{s}$ is the laser angular frequency in the steady state [20]. It can be seen the RO frequency is determined by both SL associated parameters $G_{N}$, $N_{0}$, $\tau_{s}$, $\tau_{p}$, α and its operation related parameters J, κ, τ. In following, we derive the measurement formula of α.

First, we perform a simplification for Equation (8). Limiting our treatment to practical case *κ* << 0.01 and neglecting second-order contribution, Equation (8) can be approximated as:

(9) $$\frac{f_{RO}}{f_{RO-zero}}=\;\sqrt{\frac{1+\frac{\kappa }{\tau_{in}}\tau \sqrt{1+\alpha^{2}}\cos\left(\omega_{s}\tau +\arctan\alpha \right)}{1+2\frac{\kappa }{\tau_{in}}\left[\tau_{p}\cos\left(\omega_{s}\tau \right)+\tau \cos\left(\omega_{s}\tau \right)\right]}}\;$$

In the SL with optical feedback, the external cavity length is usually L<1 m, thus $\tau_{p}<<\tau$, Equation (9) can be further approximated as

(10) $$\frac{f_{RO}}{f_{RO-zero}}=\sqrt{1-\frac{\kappa }{\tau_{in}}\tau [cos(\omega_{s}\tau )+\alpha \sin(\omega_{s}\tau )]}\;$$

Let us consider the following two special cases. Note: $f_{RO1}$ and $f_{RO2}$ are the RO frequencies in case 1 and case 2 respectively.

Case 1: with $\cos\left(\omega_{s}\tau \right)=0$, $\sin\left(\omega_{s}\tau \right)=1$, from Equation (10), we have

(11) $$\frac{f_{RO1}}{f_{RO-zero}}=\sqrt{1-\alpha \frac{\kappa \tau }{\tau_{in}}}\;$$

Case 2: with $\cos\left(\omega_{s}\tau \right)=1$, $\sin\left(\omega_{s}\tau \right)=0$, we have

(12) $$\frac{f_{RO2}}{f_{RO-zero}}=\sqrt{1-\frac{\kappa \tau }{\tau_{in}}}\;$$

Taking 1st order Taylor expansion for Equation (11), we can express the relative RO frequency difference as below:

(13) $$\frac{f_{RO1}-f_{RO-zero}}{f_{RO-zero}}=-\alpha \frac{\kappa L}{c\tau_{in}}\;$$

Similarly, Equation (12) can be rewritten as:

(14) $$\frac{f_{RO2}-f_{RO-zero}}{f_{RO-zero}}=-\frac{\kappa L}{c\tau_{in}}\;$$

Keeping an SL under same optical feedback κ, Equations (13) and (14) describe a linear relationship between the relative RO frequency difference and the external cavity length *L* respectively for the two cases. Denoting the gradients of the two lines as $S_{1}$ and $S_{2}$, α can be calculated by their ratio shown as below:

(15) $$\alpha =\frac{S_{1}}{S_{2}}\;$$

## 3. Simulation Test

To verify the proposed method by simulation, we need to obtain $f_{RO-zero}$ and the RO frequencies respectively at above two cases. All the RO related frequencies in Equation (13) and Equation (14) can be gained from the transient oscillation waveform of laser intensity [25] through solving L–K equations. 

**Figure 2:**
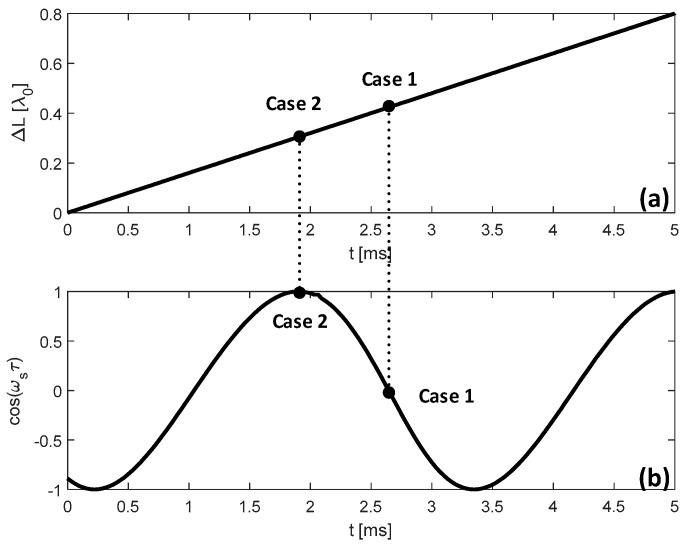
Simulation results with L=15.0 cm, κ = 0.00003, $J=1.5\;J_{\mathrm{th}}$ and α=3. (**a**): ΔL vs. Time; (**b**): An SMI signal.

**Figure 3:**
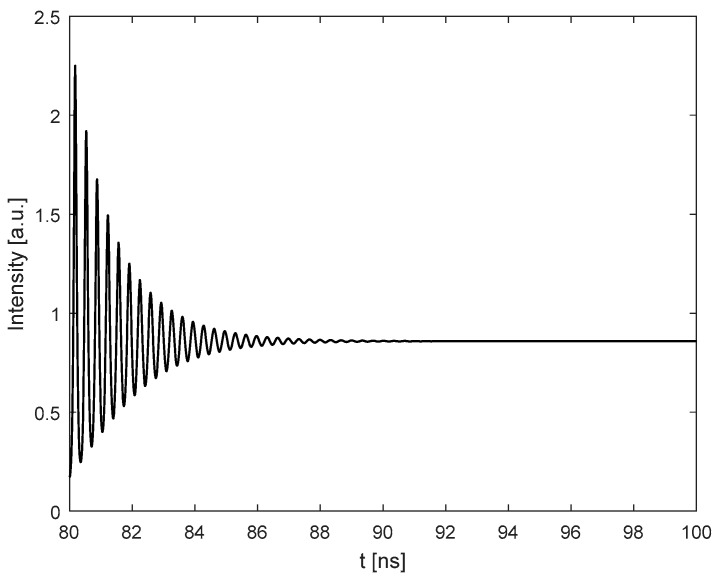
Transient waveform of the SL at Case 1 with L_0_ = 15.0 cm.

**Figure 4:**
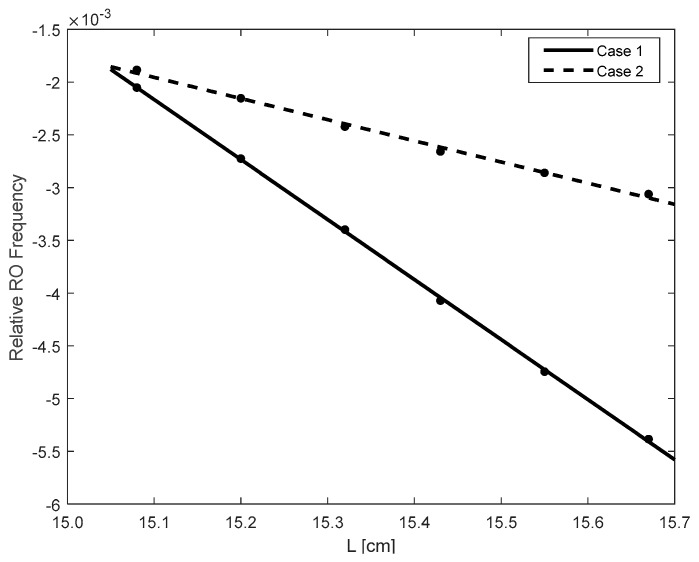
Relationship between the relative relaxation oscillation (RO) frequency difference and the external cavity length L.

The main procedure of the simulation test is summarized as below:

1. Starting from L=15.0 cm, increase the cavity length and set it with 6 different locations.
2. At each location, apply a micro displacement ΔL onto the external cavity with 0.8 wavelength shown on Figure 2a. Correspondingly, we can use the SMI model described in by Equations (4) and (7) to plot an SMI signal shown in Figure 2b, on which we can locate the accurate locations for case 1 and case 2.
3. With the results obtained at step 2 for case 1 and case 2, generate the corresponding laser intensity $E{\left(t\right)}^{2}$ by numerically solving the L-K equations. E.g., the laser transient waveform for case 1 shown in Figure 3, from which, the RO frequency can be obtained.
4. Repeat steps 2 and 3 for the 6 different locations, we get 6 RO frequencies for each case, denoted by $f_{RO1i}$ and $f_{RO2i}$, *i* = 1,2, … 6. The relationship between the relative RO frequency ($(f_{ROi}-f_{RO-zero})/f_{RO-zero}$) and the cavity length *L* for the two cases are depicted on Figure 4. Note that the gradient can be determined by only 2 points. In order to reduce the measured error, we prefer to use more than 2 points, e.g., 6 points to do line fitting to get the gradients at each case. From the gradients of these two fitting lines, α can be calculated by using Equation (15).

Figure 4 shows the simulation results when κ = 0.00003, $J=1.5\;J_{\mathrm{th}}$ with a preset α = 3. From Figure 4 we get the gradients of the two lines as *S*_1_ = 0.0050 and *S*_2_ = 0.0017, then α = 2.94 which is close to the preset value of 3. Under the same operation condition, we change the preset value of α with different values and measure it by using the above method. The results are shown in Table 2. Relative error is used to measure the measurement performance, calculated by $\left|\alpha -\hat{\alpha }\right|/\alpha$, where α is the preset true value and $\hat{\alpha }$ is calculated using the proposed method. It can be seen that the performance is satisfactory. Then we change the injection current with $J=1.3\;J_{\mathrm{th}}$, Table 3 shows the measured results with the similar relative error as in Table 2. It can be concluded that the proposed method can work for different α values including small α with α<1. This method does not need to know any internal or external parameters related to the SL, also does not need the external target having a symmetric reciprocate movement. We achieved α measurement by using RO frequencies without relying on the SMI waveform.

## 4. Experiments

**Figure 5:**
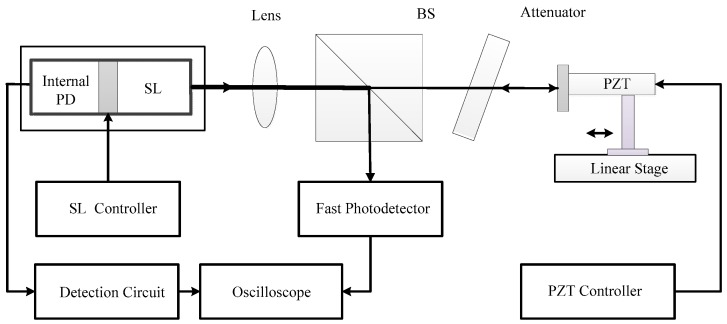
Experimental set-up. BS: beam splitter; PD: photodiode; SL: semiconductor lasers; PZT: piezoelectric transducer.

To verify the proposed method, we further built an experimental system as depicted in Figure 5. The SL in the experiment is a single mode laser diode (Sanyo, Osaka, Japan, d-001S) with a wavelength of 780 nm and maximum output power of 25 mW, which is driven and temperature-stabilized by a SL controller (Thorlabs, Newton, NJ, USA, ITC4001) at an injection current of 35 mA and at the temperature of 23±0.01 °C. The light emitted by the SL is focused by a lens and then hits the piezoelectric transducer (PZT) (Thorlabs, Newton, NJ, USA, PAS005). An attenuator is used to adjust the optical feedback strength. The PZT with a displacement resolution of 20 nm, driven by a PZT controller (Thorlabs, Newton, NJ, USA, MDT694), is used to continuously adjust the external optical phase to satisfy the requirements of case 1 and case 2 described in Section 2. The PZT is assembled on a linear translation stage to change the external cavity at different locations. The photodiode (PD) packaged at the rear of the SL is connected to a detection circuit to record an SMI signal when varying the PZT. A beam splitter (BS) with a splitting ratio of 50:50 is used to direct a part of light into the fast external photodetector (Thorlabs, Newton, NJ, USA, PDA8GS) through a fiber port coupler. This fast photodetector with a bandwidth of 9.5 GHz is suitable to capture the transient laser intensity. The SMI signals and transient laser intensity are finally captured and displayed in a fast oscilloscope (Tektronix, Beaverton, Oregon, USA, DSA 70804) with a maximum sampling rate of 25 GHz and analog bandwidth of 8 GHz. 

**Figure 6:**
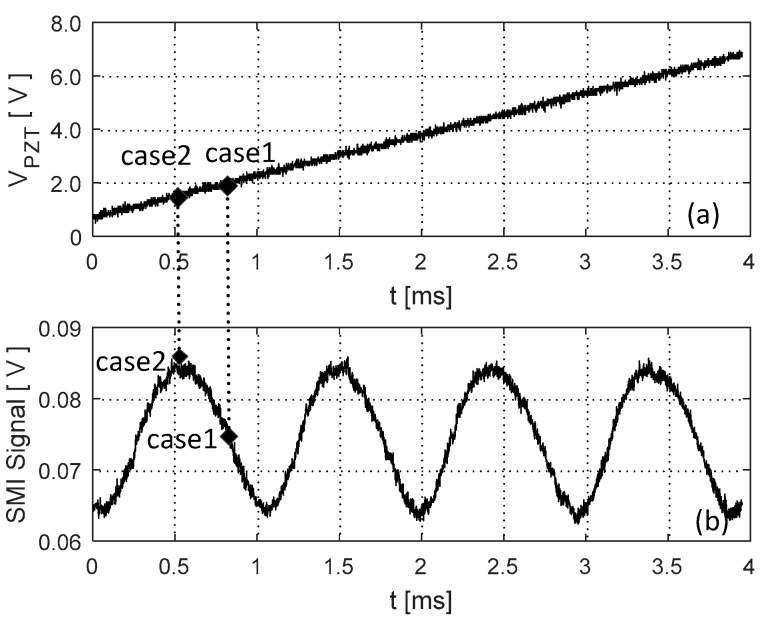
Experimental self-mixing interferometry (SMI) signal (**a**) control signal applied on PZT; (**b**) corresponding SMI signal.

**Figure 7:**
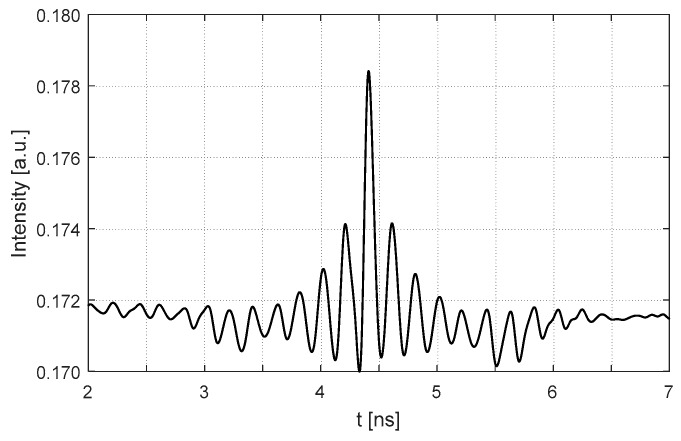
Typical laser transient intensity under quasi-continuous wave (QCW) mode.

Following the simulation test procedure described in Section 3, we choose 6 different locations for the PZT target, with the external cavity length varying from 15.0 cm to 15.5 cm. For each location, we linearly adjust the external optical phase by linearly moving the PZT with ΔL in a few wavelengths through the control voltage applied on the PZT (denoted by V_PZT_). Note that in our experiment, each 0.1 V of the V_PZT_ corresponds to 27 nm travel length of the PZT. Figure 6a shows the control signal applied on the PZT and Figure 6b is the corresponding SMI signal. After signal processing on the raw experimental signals, we are able to determine the locations for case 1 and case 2. We then set the SL working under quasi-continuous wave (QCW) mode. In this case, by using the method in [25], the transient laser intensity can be captured by using the external fast photodetector and oscilloscope. Figure 7 shows one of the experimental signals for the transient laser intensity. Still, we apply digital signal processing on the raw experimental signal and make the signal clearer. Then, the period of the transient laser intensity can be measured to get the required RO frequency.

**Figure 8:**
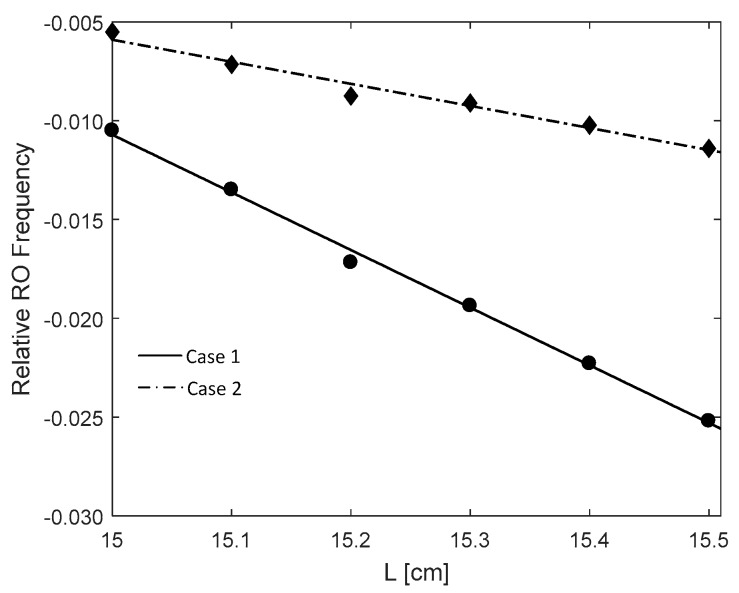
Fitting curves of the experimental results. RO: relaxation oscillation.

The experimental data and calculated result of α are presented in Table 4 where the laser injection current is 35 mA and the temperature is 23 ± 0.01 °C. The RO frequency in case 1 is denoted by $f_{RO1}$ and case 2 by $f_{RO2}$. The relationship between $f_{RO}$ and *L* for the two cases are depicted in Figure 8 with line fitting. Note, we use the relative RO frequencies difference as expressed in Equations (13) and (14), where $f_{RO-zero}=4.751\;\mathrm{GHz}$. The gradients $S_{1}$ and $S_{2}$ of the two lines are respectively 0.139 for case 1 and 0.053 for case 2. According to Equation (15), we obtain that α factor for the laser diode used in our experiment is 2.62. We also measure α factor under different laser operation condition, i.e., injection current *J* = 30 mA, *T* = 25±0.01 °C , in this case we get *α* = 2.75. Since currently, there is no a commercial measurement device for α, thus we do not have a true value of α to justify our measured value. However, we can verify our results using the method in [7]. For the same laser diode, we applied the method presented in [7] with *J* = 35 mA, T = 23 ± 0.01 °C , *L*_0_ = 15 cm under moderate feedback regime, it gives *α* = 2.89 which is close to the result obtained by the proposed method.

## 5. Conclusions

The RO frequency of a laser can be modified by external optical feedback. Based on this fact, we investigated the relation between the RO frequency and α factor and presented a new method for measuring this factor. The proposed method is verified by simulations using L–K equations. It is also confirmed with the experiments and compared the result obtained with other reported method [7]. This work has the advantage that it does not need to know any parameters related to internal or external parameters associated to the laser and not rely on the SMI waveform. In addition, this method can work for the case with α less than 1 and does not need the external target having a symmetric reciprocate movement. Furthermore, as the RO can be observed in many types of lasers, the proposed method is not limited to semiconductor lasers. 

## Figures and Tables

**Table 1:** Physical meaning of symbols in Equations (1)–(3).

Symbol	Physical Meaning	Value
κ	Feedback strength	
τ	External cavity round trip time,τ=2L/c, where *L* is external cavity length, *c* is speed of light	
$\omega_{0}$	Angular frequency of solitary laser	
α	Line-width enhancement factor	
J	Injection current density	
$J_{\mathrm{th}}$	Threshold injection current density	
$\tau_{in}$	Internal cavity round-trip time	$8.0\times 10^{-12}\;s$
$\tau_{p}$	Photon life time	$2.0\times 10^{-12}\;s$
$\tau_{s}$	Carrier life time	$2.0\times 10^{-9}\;s$
$G_{N}$	Modal gain coefficient	$8.1\times 10^{-13}\;m^{3}s^{-1}$
$N_{0}$	Carrier density at transparency	$1.1\times 10^{24}\;m^{-3}$
ε	Nonlinear gain compression coefficient	$2.5\times 10^{-23}\;m^{3}$
Γ	Confinement factor	0

**Table 2:** Simulation results with κ = 0.00003, $J=1.5\;J_{\mathrm{th}}$.

α(τρυε)	$\hat{\alpha }\left(simulated\right)\;$	Error %
0.50	0.52	3.8%
1.00	1.01	0.6%
2.00	2.04	1.9%
3.00	2.94	2.0%
4.00	3.81	4.8%
5.00	4.75	5.0%

**Table 3:** Simulation results with κ = 0.00003, $J=1.3\;J_{\mathrm{th}}$.

α(true)	$\hat{\alpha }\left(simulated\right)\;$	Error %
0.50	0.51	2.4%
1.00	1.04	4.2%
2.00	2.05	2.7%
3.00	3.08	2.7%
4.00	4.01	0.2%
5.00	4.82	3.7%

**Table 4:** Experimental results.

L (cm)	15.0	15.1	15.2	15.3	15.4	15.5
$f_{RO1}\;(\mathrm{GHz})$	4.631	4.645	4.659	4.669	4.687	4.701
$f_{RO2}\;(\mathrm{GHz})$	4.697	4.702	4.708	4.709	4.717	4.725
$f_{RO-zero}\;(\mathrm{GHz})$	4.751					
$S_{1}=0.139$, $S_{2}=0.053$, α=2.62.						
